# Transcriptomic Changes of *Bemisia tabaci* Asia II 1 Induced by Chilli Leaf Curl Virus Trigger Infection and Circulation in Its Vector

**DOI:** 10.3389/fmicb.2022.890807

**Published:** 2022-04-28

**Authors:** Aarthi Nekkanti, Prosenjit Chakraborty, Amalendu Ghosh, Mir Asif Iquebal, Sarika Jaiswal, Virendra Kumar Baranwal

**Affiliations:** ^1^Advanced Centre for Plant Virology, Indian Agricultural Research Institute, New Delhi, India; ^2^Division of Entomology, Indian Agricultural Research Institute, New Delhi, India; ^3^Centre for Agricultural Bioinformatics, Indian Agricultural Statistics Research Institute, New Delhi, India

**Keywords:** ChiLCV, silverleaf whitefly, RNA-Seq, transcriptome, RT-qPCR, virus-vector relationship

## Abstract

*Bemisia tabaci* (Hemiptera: Aleyrodidae) is a highly efficient vector in the spread of chilli leaf curl virus (ChiLCV, *Begomovirus*) which is a major constraint in the production of chilli in South Asia. Transcriptome analysis of *B. tabaci* post-6 h acquisition of ChiLCV showed differential expression of 80 (29 upregulated and 51 downregulated) genes. The maximum number of DEGs are categorized under the biological processes category followed by cellular components and molecular functions. KEGG analysis of DEGs showed that the genes are involved in the functions like metabolism, signaling pathways, cellular processes, and organismal systems. The expression of highly expressed 20 genes post-ChiLCV acquisition was validated in RT-qPCR. DEGs such as cytosolic carboxypeptidase 3, dual-specificity protein phosphatase 10, 15, dynein axonemal heavy chain 17, fasciclin 2, inhibin beta chain, replication factor A protein 1, and Tob1 were found enriched and favored the virus infection and circulation in *B. tabaci*. The present study provides an improved understanding of the networks of molecular interactions between *B. tabaci* and ChiLCV. The candidate genes of *B. tabaci* involved in ChiLCV transmission would be novel targets for the management of the *B. tabaci*-begomovirus complex.

## Introduction

Transmission of a plant virus within or between the fields is often dependent upon a mobile vector. Insects are the most efficient vectors of plant viruses because of their abundance and feeding behavior (Milenovic et al., 2019). About 70% of reported insect vectors are hemipterans (Fereres and Raccah, 2015). Silverleaf whitefly, *Bemisia tabaci* Gennadius (Hemiptera: Aleyrodidae) is an invasive insect pest and vector of several plant viruses (Legarrea et al., 2015). Transmission of several begomoviruses, carlaviruses, criniviruses, cytorhabdoviruses, ipomoviruses, poleroviruses, and torradoviruses by *B. tabaci* causes economic losses exceeding billions of US$ and threatens food security (Dasgupta et al., 2003; Jones, 2003; Navas-Castillo et al., 2011; Brown et al., 2015; Orfanidou et al., 2016; Saeed and Samad, 2017; Zanardo and Carvalho, 2017; Ghosh et al., 2019; Costa et al., 2020; Pinheiro-Lima et al., 2020; Cornejo-Franco et al., 2022).

The spectrum of diseases caused by begomoviruses is a continuing challenge to crop production worldwide (family: *Geminiviridae*) (Navas-Castillo et al., 2011). These diseases cause an estimated yield loss of 50–90% in tomatoes, chilli, and other crops including beans, cassava, cotton, cucurbits, eggplant, papaya, and potatoes (Briddon et al., 2010; Saeed and Samad, 2017). To date, 445 begomovirus species have been reported^1^. One of the begomoviruses, i.e., chilli leaf curl virus (ChiLCV) is a major threat to chilli production in tropical and sub-tropical countries (Senanayake et al., 2007, 2012; Menike and De Costa, 2017; Oraonand and Tarafdar, 2018; Thakur et al., 2018). ChiLCV has caused several epidemics in India and Sri Lanka (Senanayake et al., 2007, 2012). The disease is typically manifested in the infected plants as upward curling, puckering, and bunching of leaves. The leaves become smaller and severely affected plants produce fewer and deformed fruits. Yield loss of 20–50% has been recorded in chilli by ChiLCV (Thakur et al., 2018) which may rise to 100% with the infestation of thrips and mites (Menike and De Costa, 2017). Control options for *B. tabaci*-ChiLCV are very limited as insecticides continue to lose their efficacy due to the emergence of resistant *B. tabaci* populations (Barman et al., 2021). Additionally, insecticides adversely affect the environment and human health. Understanding the molecular interactions between *B. tabaci* and ChiLCV and interrupting the interrelationship is a promising approach to manage the virus-vector complex. The present understanding of *B. tabaci*-begomovirus interaction is largely based on the study of *B. tabaci* cryptic species MEAM1 and MED and tomato yellow leaf curl virus (TYLCV). Once ingested by *B. tabaci*, virus particles pass through the midgut, where they move across the midgut membrane into the hemolymph, possibly *via* receptor-mediated endocytosis, and circulate to the primary salivary glands, from where they are egested along with saliva during feeding (Czosnek and Ghanim, 2012). During the entire process, the viral proteins need to interact with several proteins in the midgut, hemolymph, and primary salivary glands (Wei et al., 2014). *B. tabaci* MEAM1 heat shock proteins (Hsp), cyclophilins, and peptidoglycan recognition protein interact with TYLCV coat protein (CP) for successful internalization (Götz et al., 2012; Kanakala and Ghanim, 2016; Wang et al., 2016). Silencing of *hsp70* in *B. tabaci* Asia II 1 inhibits transmission of ChiLCV (Chakraborty and Ghosh, 2022). A GroEL homolog produced by C-type endosymbionts in *B. tabaci* MEAM1 is known to transport TYLCV particles through the hemolymph of *B. tabaci* in coated vesicles (Bragard et al., 2013).

Over the past 5 years, transcriptomic analysis of *B. tabaci* has enabled us to study the differential expression of genes that are involved in virus transmission (Luan et al., 2011; Kaur et al., 2017; Xia et al., 2017; Hasegawa et al., 2018; Ding et al., 2019). However, these studies were limited to gene regulations in MEAM1 and MED cryptic species of *B. tabaci* in response to infection of TYLCV, tomato yellow leaf curl China virus (TYLCCV), and a crinivirus-tomato chlorosis virus (ToCV). To date, 46 morphologically indistinguishable cryptic species of *B. tabaci* are known (Rehman et al., 2021) that differ in genetic structure, chemical resistance, adaptability, and virus transmission (Brown, 2000; Gorman et al., 2010; Wang et al., 2010; Qin et al., 2016). The genes of *B. tabaci* involved in the transmission of begomoviruses are not conserved across all the *B. tabaci* cryptic species–begomovirus combinations. Also, little is known about the role of putative genes of *B. tabaci* Asia II 1 in response to ChiCLV transmission. The present study reports the candidate genes of *B. tabaci* Asia II 1 that are regulated at an early stage of ChiLCV infection. Identification of candidate genes involved in key physiological processes and ChiLCV infection would be novel targets for the management of the *B. tabaci*- ChiLCV complex.

## Materials and Methods

### Establishment of Isofemale Population of *Bemisia tabaci*

A homogeneous population of *B. tabaci* Asia II 1 maintained at Advanced Centre for Plant Virology, Indian Agricultural Research Institute (IARI), New Delhi since 2015 was used in the present study. The homogeneous population was developed from a single adult female on healthy eggplants. The cryptic identity of the *B. tabaci* was confirmed by PCR amplification of the mitochondrial cytochrome oxidase subunit I (mtCOI) using C1-J-2195 and L2-N-3014 primers (Simon et al., 1994; Supplementary Table 1) and sequencing. DNA was isolated from randomly collected adult flies from the homogeneous population using CTAB extraction buffer as described by Rehman et al. (2021). PCR was performed in a 25 μl reaction mixture comprised of 1x DreamTaq buffer, 0.1 mM dNTPs (Thermo Fisher Scientific, Waltham, MA, United States), 10 picomoles forward and reverse primers, 1.25 U DreamTaq DNA polymerase (Thermo Fisher Scientific, Waltham, MA, United States), and 50 ng of template DNA. Thermal cycling was followed as initial denaturation at 94°C for 2 min, 30 cycles of denaturation at 94°C for 30 s, annealing at 53°C for 30 s and polymerization at 72°C for 1 min, followed by final extension step at 72°C for 10 min. PCR products were visualized on 1% agarose gel stained with GoodView™ (BR Biochem, New Delhi, India). The purified PCR products were sequenced bidirectionally. The sequences were processed by BioEdit and species homology was checked in BLASTn. A consensus sequence was submitted to GenBank. The genotype or cryptic species of the *B. tabaci* population was confirmed based on Bayesian Inference phylogeny considering a genetic divergence cutoff of 4% as described by Rehman et al. (2021). The population was maintained under controlled environmental conditions at 28 ± 2°C, 60 ± 10% relative humidity, and 16 h light- 8 h dark photoperiod.

### Virus Isolate and Generation of ChiLCV-Exposed and Non-exposed *Bemisia tabaci*

A pure culture of ChiLCV maintained on chilli plant (var. Preeti, Nunhems, Haelen, Netherlands) by *B. tabaci* inoculation under insect-proof conditions was used in the study. ChiLCV was confirmed by amplifying DNA-A using Begomo F and Begomo R primers (Akhter et al., 2009) (Supplementary Table 1) and nucleotide sequencing. To establish the ChiLCV-exposed and non-exposed *B. tabaci* populations, freshly emerged female adults were collected and released onto ChiLCV-infected and virus-free chilli plants at 4–6 leaf stage for 6 h in three biological replicates. A 6 h acquisition was found adequate for the successful transmission of ChiLCV by *B. tabaci* Asia II 1 (Senanayake et al., 2012; Roy et al., 2021). After 6 h of acquisition, ChiLCV-exposed (BtTrI1, BtTrI2, and BtTrI3) and non-exposed (BtTrH1, BtTrH2, and BtTrH3) *B. tabaci* adults were collected. ChiLCV infection in *B. tabaci* populations was confirmed by randomly collecting 10 flies from each population and testing in PCR using ChiLCV-specific primers (AG149F-AG150R) (Roy et al., 2021, Supplementary Table 1). Each population was divided into two parts. One part was utilized for RNA-Seq and another part was preserved in −80°C for gene expression analysis in a reverse transcription-quantitative polymerase chain reaction (RT-qPCR).

### Total RNA Isolation

Total RNA was isolated from each ChiLCV-exposed (BtTrI1, BtTrI2, and BtTrI3) and non-exposed (BtTrH1, BtTrH2, and BtTrH3) *B. tabaci* populations using Trizol reagent (Invitrogen, Waltham, MA, United States) following manufacturer’s protocol. In brief, 50 *B. tabaci* were crushed in 1 mL Trizol and kept at room temperature for 10 min. Two hundred microliter of chloroform was added to the mixture, vortexed for <10 s, and incubated at room temperature for 10 min. The mixture was then centrifuged at 16,000 x*g* for 10 min at 4°C. The upper aqueous phase was transferred to a fresh tube and 0.8 volume of ice-chilled isopropanol was added, mixed properly, and incubated at 4°C for 10 min. The mixture was again centrifuged at 16,000 x*g* for 10 min at 4°C and the supernatant was discarded. The pellet was finally washed with 70% ethanol, air-dried, and resuspended in 30 μl nuclease-free water. RNaseZAP (Thermo Fisher Scientific, Waltham, MA, United States) was used for decontamination, RNase-free tips, microfuge tubes, and water were used throughout the experiment. Total RNA quality was measured using RNA 6000 Nano Kit (Agilent Technologies, Santa Clara, CA, United States) on 2100 Bioanalyzer (Agilent Technologies) with a minimum RNA Integrity Number (RIN) value of 7. RNA concentrations were determined using a NanoDrop ND-8000 spectrophotometer (Thermo Fisher Scientific, Waltham, MA, United States).

### Construction of cDNA Library and Sequencing of Transcripts

RNA-Seq libraries for all samples were prepared using NEBNext UltraII RNA library preparation kit for Illumina (New England Biolabs, Ipswich, MA, United States) following manufacturer’s protocol and sequencing was done in a single HiSEQ 4000 (Illumina Inc., San Diego, CA, United States) lane using 150 bp paired-end chemistry. The library preparation and sequencing were done by commercial service providers (NxGenBio Life Sciences, Delhi, India). Briefly, mRNA was purified using oligo-dT beads. Magnetic beads were used for the second round of purification. During the second elution of the poly-A RNA, the RNA was also fragmented into short stretches of 200–500 bp at 94°C for 5 min using an ultrasonicator in presence of divalent cations. The cleaved RNA fragments were copied into first-strand cDNA using SuperScript-II reverse transcriptase (Thermo Fisher Scientific, Waltham, MA, United States) with random primers. After second-strand cDNA synthesis, fragments were end-repaired, A-tailed, and ligated to indexed adapters. The products were purified and enriched with PCR to create the final cDNA library. The tagged cDNA libraries were pooled in equal ratios and used for 2 × 150 bp paired-end sequencing on a single lane of the Illumina HiSEQ 4000. Illumina clusters were generated and were loaded onto Illumina Flow Cell and sequenced. After sequencing, the samples were demultiplexed and the indexed adapter sequences were trimmed using the CASAVA v1.8.2 software (Illumina Inc.).

### Pre-processing of Raw Reads and Differential Gene Expression Analysis

The ambiguous “N” nucleotides with a ratio of “N” > 5%, reads with adaptor sequences, and low-quality reads with a quality score < 20% were removed by the Trim Galore v0.4.1 to get the high-quality reads. Reference genome index was established using BWAv0.7.5 and the clean reads were mapped to the reference genome of *B. tabaci* MEAM1 (Chen et al., 2016). Read numbers, mapped to every gene were counted using Samtools v0.1.19. Differential expression between the ChiLCV-exposed (BtTrI1, BtTrI2, and BtTrI3) and non-exposed (BtTrH1, BtTrH2, and BtTrH3) *B. tabaci* populations was analyzed using the DESeq R package^2^. Significant differential gene expression that was consistent among the biological replicates, was counted with ≥ log_2_ 2-fold change and *p* < 0.05.

### Annotation and Functional Enrichment Analysis of Differentially Expressed Genes

Gene annotations and functional enrichment analysis including Gene Ontology (GO) and Kyoto Encyclopedia of Genes and Genomes (KEGG) biological pathways were used to identify the differentially expressed genes (DEGs) that were significantly enriched in GO terms or biological pathways post-6 h of ChiLCV acquisition. Gene annotations against the Uniprot GO database^3^ were performed by aligning DEGs to the NR database using the blast v 2.6.0+ programme. KEGG pathway enrichment analysis of DEGs was performed using the KEGG database resource^4^ by KAAS (Moriya et al., 2007) to identify the pathways that were differentially regulated between ChiLCV-exposed and non-exposed *B. tabaci* with *p*-value < 0.05.

### Relative Expression of Putative Genes in Reverse Transcription-Quantitative Polymerase Chain Reaction

To validate the RNA-Seq data, 20 putative genes of *B. tabaci* Asia II 1 with ≥log_2_ 2-fold change values were selected to assess the gene expression in RT-qPCR. Two sets of primers were designed for each of the target and endogenous control (β-actin) genes. The primer pairs were initially optimized in a gradient PCR. One set of primers for each target gene was selected based on the efficiency of PCR amplification at the same PCR conditions for the endogenous control primers. A part of the same ChiLCV-exposed and non-exposed *B. tabaci* populations used for RNA-Seq was preserved for RT-qPCR analysis. Total RNA was isolated from ChiLCV-exposed (BtTrI1, BtTrI2, and BtTrI3) and non-exposed (BtTrH1, BtTrH2, and BtTrH3) *B. tabaci* populations as described earlier. cDNA was synthesized using the FIREScript RT cDNA synthesis kit (Solis BioDyne, Estonia). The reaction mixture contained 1X RT reaction buffer, 1.0 μg template RNA, 5.0 μM oligo dT primer, 500 μM dNTP mix, 10-unit FIREScript RT, and 1-unit RiboGrip RNase inhibitor. The reverse transcription was carried out in a thermocycler (T100, Bio-Rad, Hercules, CA, United States) at 42°C for 60 min followed by enzyme inactivation at 85°C for 5 min.

The relative RT-qPCR was carried out in an InstaQ 48 real-time PCR (Himedia, Mumbai, India) with 20 μl reaction volume containing 10 μl of 2X Maxima SYBR green master mix, 10 μM ROX passive reference dye, 10 picomoles each forward and reverse primer (Supplementary Table 1), and 2 μl template cDNA. Thermal cycling was performed as initial denaturation at 94°C for 5 min, 30 cycles of 94°C for 30 sec, 56°C for 30 sec, and 72°C for 30 sec. The dissociation or melting stage was carried out after every reaction to determine the specificity of the amplicons in RT-qPCR using a computer interface programme for InstaQ 48M2 (Himedia). The RT-qPCR was performed with three biological and two technical replicates. The expression change of the target gene was normalized by excluding the changes in cycle threshold (C_*T*_) value of endogenous control, β-actin. Log_2_-fold change value was calculated and relative expression of mRNA was determined by normalizing the log_2_ values of the ChiLCV-exposed populations with non-exposed using the 2^–ΔΔ^ *^C^*_*T*_ method (Livak and Schmittgen, 2001) in Mircosoft Excel 2016.

## Results

### Characterization of *Bemisia tabaci* and Begomovirus

An isofemale line of *B. tabaci* Asia II 1 (Accession No. MT920041), maintained on healthy eggplant at Advanced Centre for Plant Virology, IARI was used to generate ChiLCV-exposed and non-exposed *B. tabaci* populations. The identity of the *B. tabaci* cryptic species was confirmed by sequencing the mtCOI gene. PCR amplification of *B. tabaci* mtCOI with C1-J-2195 and L2-N-3014 primers showed an expected amplicon of ∼860 bp on an agarose gel. The nucleotide (nt) sequence showed 99.99% homology in BLASTn analysis with other *B. tabaci* sequences in NCBI. The sequence can be retrieved using the GenBank Accession No. MT920041. Bayesian Inference phylogeny considering genetic divergence cutoff of 4% revealed that the population belonged to the cryptic species *B. tabaci* Asia II 1 (data not shown).

PCR amplification of the DNA-A using Begomo F and Begomo R primer produced a 2.7 kb product as visualized on 1% agarose gel. Bidirectional sequencing of the cloned products produced an 1896 nt sequence, comprising complete AV1 and AV2 genes, and partial AC1, AC2, AC3, and AC4 genes, that was 100% similar to ChiLCV isolates upon BLASTn analysis. The sequence can be retrieved by GenBank Accession No. MW399222.

### ChiLCV-Exposed and Non-exposed *Bemisia tabaci* Population

ChiLCV-exposed and non-exposed *B. tabaci* populations were developed by allowing the freshly emerged adult flies to feed on infected and healthy chilli plants for 6 h. A few randomly collected *B. tabaci* adults were tested in PCR with ChiLCV-specific primers, AG149F and AG150R (Supplementary Table 1). A product of 290 bp was visualized on agarose gel that confirmed the virus infection in ChiLCV-exposed *B. tabaci* adults. *B. tabaci* populations (BtTrH1, BtTrH2, and BtTrH3) that were exposed to healthy chilli plants did not produce any ChiLCV-specific amplification in PCR.

### Illumina Sequencing and Assembly

Total RNA was extracted from three ChiLCV-exposed (BtTrI1, BtTrI2, and BtTrI3) and non-exposed (BtTrH1, BtTrH2, and BtTrH3) *B. tabaci* Asia II 1 populations. For RNA-Seq analysis of ChiLCV-exposed and non-exposed *B. tabaci*, a total of six cDNA libraries were constructed and used for 2 × 150 bp pair-end sequencing on a single HiSEQ4000 lane. About 26–33 million raw reads were generated from each library (Table 1). Of which, 99.58–99.70% reads were clean reads. The cleaned reads of all the six libraries were mapped with the reference genome of *B. tabaci* MEAM1 (Chen et al., 2016). The mapping percent for all six libraries ranged from 87.21 to 93.16% of clean reads.

**TABLE 1:** Summary of RNA-Seq data obtained from ChiLCV-exposed and non-exposed *B. tabaci* Asia II 1.

Sample		Raw reads	Filter reads	Clean reads (%)	% mapped with reference genome
ChiLCV non-exposed *B*. *tabaci*	BtTrH1	2,65,84,539	2,65,09,920	99.7	91.7
	BtTrH2	2,65,05,012	2,63,95,200	99.58	87.38
	BtTrH3	3,12,05,804	3,11,08,963	99.68	87.21
ChiLCV exposed *B. tabaci*	BtTrI1	3,31,48,645	3,30,35,921	99.65	93.16
	BtTrI2	2,97,68,269	2,96,75,113	99.68	88.01
	BtTrI3	2,67,77,701	2,66,83,511	99.64	90.03

### General Pattern of *Bemisia tabaci* Gene Expression in Response to ChiLCV Infection

**FIGURE 1:**
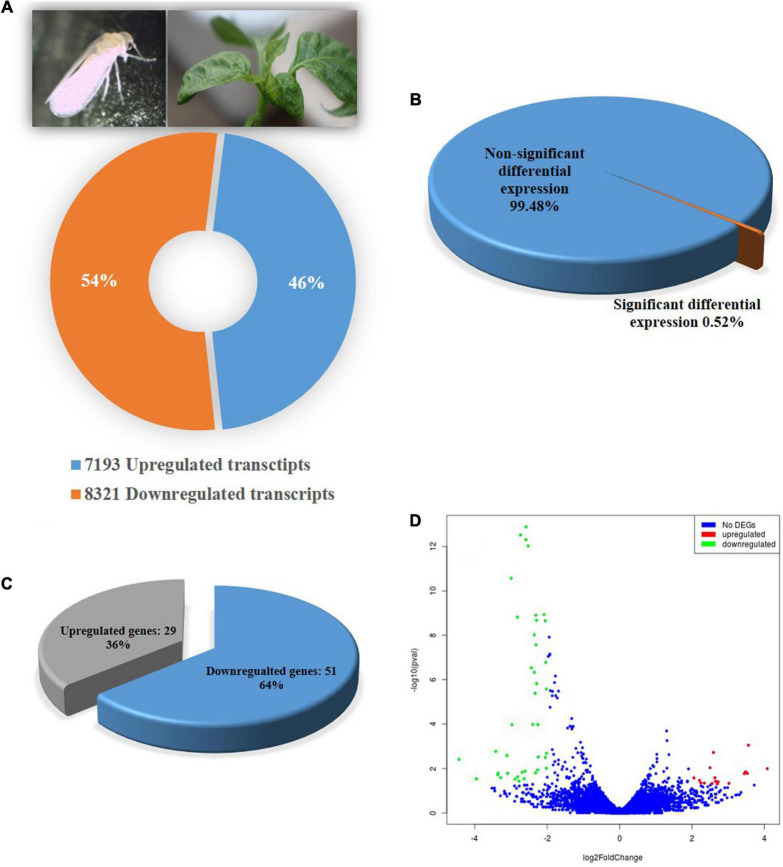
Differential gene expression of *B. tabaci* Asia II1 in response to ChiLCV infection. **(A)** Percentage of upregulated and down-regulated transcripts; **(B)** Percentage differentially expressed genes of *B. tabaci* at the expression level of ≥log_2_ 2-fold and *p*-value ≤ 0.05; **(C)** Proportion of differentially expressed genes (DEGs) in RNA-Seq analysis. A total of 29 DEGs were found upregulated while 51 were downregulated; **(D)** Volcano plot of DEGs. The x-axis shows the fold change in gene expression between different samples, and the y-axis shows the statistical significance of the differences. Significantly up- and downregulated genes with log_2_ FC ≥ 2 are highlighted in red and green, respectively. The blue dots represent non-significant DEGs.

A total of 15,514 genes in adult *B. tabaci* Asia II 1 were found to be differentially expressed post 6 h of ChiLCV exposure. Out of which, 7,193 genes were upregulated and 8,321 genes were downregulated. However, only a total of 80 (0.52%) genes showed significant regulations with ≥log_2_ 2-fold change in expression level at a significant *p*-value of ≤0.05. Among the significant differentially expressed genes (DEGs), 29 genes were upregulated and 51 genes were downregulated in combined BtTrI as compared to BtTrH (Figure 1). The top upregulated and downregulated genes of *B. tabaci* in response to ChiLCV-infection are listed in Table 2. The DEGs of *B. tabaci* were involved in receptor binding, antigen binding, epithelial cell differentiation, extracellular matrix organization, cell-to-cell, and cell-surface receptor signaling. Besides, a large number of orphan genes were significantly regulated in *B. tabaci* post 6 h of ChiLCV acquisition.

**TABLE 2:** Differentially expressed upregulated and downregulated genes of *B. tabaci* Asia II 1 in response to ChiLCV infection.

Sl No.	Gene ID	Gene name	GO term	Log_2_ fold change	*P*-value
**Upregulated genes**					
1	Bta12916	Toll receptor 3	Involved in control host immune response, activated by double-stranded RNA, a sign of viral infection	3.6	0.0009
2	Bta06925	Cytosolic carboxypeptidase 3	Catalyzes the deglutamylation of polyglutamate side chains generated by post-translational polyglutamylation in proteins such as tubulins	2.6	0.001
3	Bta15365	Dynein heavy chain	Involved in ATPase activity, plays a major role in sperm motility, implicated in sperm flagellar assembly and beating	4.1	0.01
4	Bta07280	Tob1	Plays an important role in controlling cell cycle progression, suppressing tumor development	3.4	0.01
5	Bta00655	GMP reductase	Functions in the conversion of nucleobase, nucleoside, and nucleotide derivatives of G to A nucleotides, and in maintaining the intracellular balance of A and G nucleotides	3.5	0.014
6	Bta13317	Replication factor A	Plays an essential role in DNA replication, recombination, and repair. Binds and stabilizes single-stranded DNA intermediates	3.5	0.016
7	Bta00469	Fasciclin 2	Homophilic cell adhesion *via* plasma membrane adhesion molecule, synapse organization	2.6	0.02
8	Bta06684	Estrogen sulfotransferase	Catalyzes the sulfate conjugation of many hormones, neurotransmitters, drugs, and xenobiotic compounds	2.3	0.04
9	Bta02631	Deoxyribonuclease I	Cleaves protein-free DNA, involved in cell death by apoptosis. Together with DNASE1L3, plays a key role in degrading neutrophil extracellular traps	2.2	0.04
10	Bta07061	Unknown protein	-	2.5	0.009
11	Bta03305	Unknown protein	-	2.1	0.02
12	Bta15645	Unknown protein	-	2.7	0.03
13	Bta12076	Unknown protein	-	2.6	0.03
14	Bta09526	Unknown protein	-	2.2	0.034
15	Bta08486	Unknown protein	-	3	0.04
16	Bta10794	Unknown protein	-	2.7	0.04
17	Bta11684	Unknown protein	-	2.6	0.04
**Downregulated genes**					
18	Bta00788	Protein argonaute 2	RNA interference (RNAi) pathway. A member of the RNA-induced transcriptional silencing (RITS) complex	−2.6	1.30E-13
19	Bta09958	Protein argonaute 2	Involved in RNA interference (RNAi) pathway. A member of the RNA-induced transcriptional silencing (RITS) complex	−3	2.70E-11
20	Bta07464	Pupal cuticle protein 36	Component of the pupal abdominal endocuticle. May have an important role in the larval and adult exoskeleton structure.	−2.6	2.54E-09
21	Bta05467	Major royal jelly protein	Induces the differentiation of honeybee larvae into queens through an Egfr-mediated signaling pathway	−3	0.0001
22	Bta10408	Prolyl 4-hydroxylase alpha-1 subunit	Iron ion binding, L-ascorbic acid-binding, procollagen proline 4 dioxygenase activity	−3.1	0.01
23	Bta13082	Endochitinase A	Involved in cortical microtubule organization	−2.6	0.013
24	Bta15272	Inhibin beta chain	Germ cell development and maturation, nerve cell survival, embryonic axial development	−2.7	0.014
25	Bta05443	T-box transcription factor TBX20	Acts as a transcriptional activator and repressor required for cardiac development and may have key roles in the maintenance of functional and structural phenotypes in adult heart	−3.4	0.016
26	Bta10856	Protein phosphatase 1L	Acts as a suppressor of the SAPK signaling pathways by associating with and dephosphorylating MAP3K7/TAK1 and MAP3K5, and by attenuating the association between MAP3K7/TAK1 and MAP2K4 or MAP2K6.	−3.4	0.019
27	Bta07414	Neurobeachin-like protein 1	Protein kinase binding and protein localization	−3.3	0.02
28	Bta01185	Anther-specific proline-rich protein	Serine type endopeptidase involved in inhibitor activity	−2.8	0.02
29	Bta03315	Adenosine deaminase	Plays an important role in purine metabolism and adenosine homeostasis	−2.6	0.028
30	Bta01284	AGAP004475-PA	Unreviewed	−4	0.029
31	Bta03804	AT-rich interactive domain-containing protein	Transcription factor which may be involved in the control of cell cycle progression by the RB1/E2F1 pathway and in B-cell differentiation	−2.9	0.03
32	Bta11712	Klingon	Axon guidance receptor activity, hemophilic cell adhesion *via* plasma membrane adhesion molecule	−2.8	0.03
33	Bta14472	Homeobox protein Hox-A2	Play a crucial role in several biological processes like control of cell identity, cell growth, and differentiation, cell to cell, and cell to extracellular matrix interactions	−2.2	0.01
34	Bta00780	Unknown protein	-	−2.7	3.02E-13
35	Bta04905	Unknown protein	-	−2.8	1.53E-09
36	Bta00785	Unknown protein	-	−3.4	0.001
37	Bta09990	Unknown protein	-	−4.4	0.003

### Differentially Expressed Genes in *Bemisia tabaci* Post-ChiLCV Acquisition

A total of 16 known genes were found to be upregulated in adult *B. tabaci* at an early stage of ChiLCV infection. The key upregulated genes included dual-specificity protein phosphatase 10 (DUSP10)-like, dual-specificity protein phosphatase 15 (DUSP15)-like, estrogen sulfotransferase (Ste), cytosolic carboxypeptidase 3 (AGBL3)-like, fasciclin 2 (Fas2), Tob1, GMP reductase 1 (GMPR), dentin sialophosphoprotein (DSPP), toll receptor 3 (TLR3), dynein axonemal heavy chain 17 (DNAH17), ATP-dependent DNA helicase (DDX11), WAS/WASL-interacting protein family member 1 (WIPF1), proline-rich extensin protein EPR1 (EPR1), CG13607, and glutamyl-tRNA(Gln) amidotransferase subunit A (QRSL1).

Twenty-three genes were significantly downregulated in *B. tabaci* adults post 6 h of ChiLCV acquisition. Putative genes of *B. tabaci* such as nose resistant to fluoxetine protein 6 (nrf-6), protein masquerade (mas), protein argonaute 2 (AGO2), endochitinase A (CHIA), pupal cuticle protein 36 (PCP36), keratinocyte proline-rich protein (KPRP), adenylate cyclase, juvenile hormone acid O-methyltransferase (jhamt), T-box transcription factor TBX20 (TBX20), 1-acyl-sn-glycerol-3-phosphate acyltransferase (plsC), AGAP004475-PA, protein phosphatase 1L (PPM1L), neurobeachin-like protein 1 (NBEAL1), major royal jelly protein (MRJP), prolyl 4-hydroxylase alpha-1 subunit (P4HA1), AT-rich interactive domain-containing protein (ARID), Klingon (klg), inhibin beta chain (Actbeta), adenosine deaminase (ADA), chloroquine resistance marker protein, leptin receptor (LEPR), and homeobox protein Hox-A2 (HOXA2) could be seen significantly downregulated in response to ChiLCV infection.

### Differentially Expressed Orphan Genes in *Bemisia tabaci* Post-ChiLCV Acquisition

Among the 80 differentially expressed transcripts, 50 (16 upregulated and 34 downregulated) transcripts could not be annotated based on nucleotide similarity against *B. tabaci* genome database^5^. When searched for nucleotide similarity at NCBI RefSeq non-redundant protein database, 43 (16 upregulated and 27 downregulated) transcripts did not show any similarity to known proteins. Out of the 16 upregulated genes, 9 genes showed similarity with unknown protein-coding genes. Whereas, 8 genes out of 27 downregulated genes showed similarity with unknown protein-coding genes. In common, 13 upregulated and 22 downregulated transcripts in adult *B. tabaci* could not be assigned any annotation. These genes were depicted as orphan genes that were differentially expressed in response to ChiLCV infection.

### Functional Analysis of Differentially Expressed Genes

**FIGURE 2:**
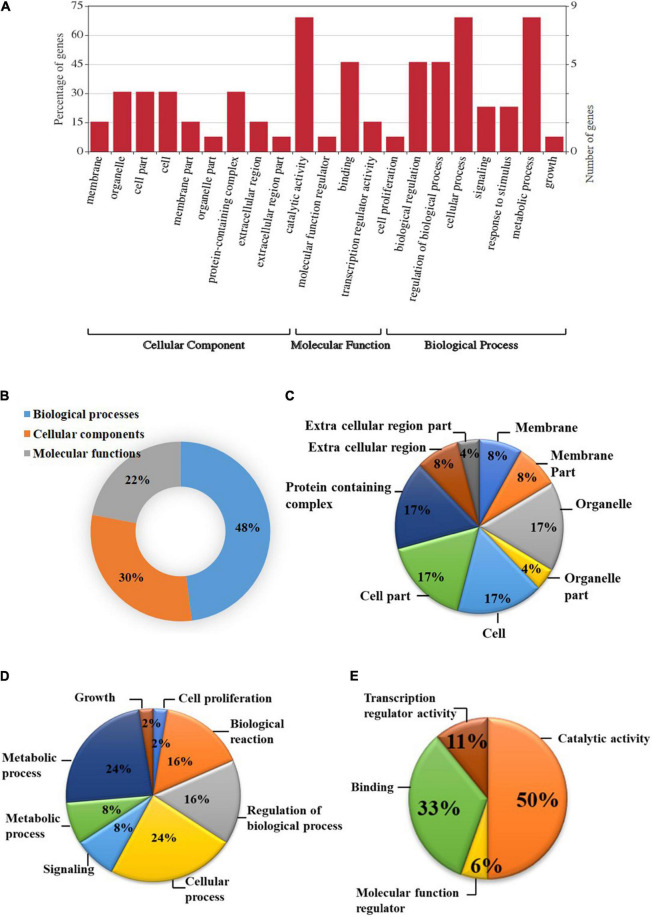
Gene ontology (GO) enrichment analysis of differentially expressed genes (DEGs). **(A)** DEGs were characterized under the cellular components, molecular functions, and biological processes based on GO analysis; **(B)** Pie chart describing the Gene Ontology (GO) analysis of differentially expressed genes (DEGs) and their distribution under different GO terms. DEGs were categorized majorly under biological processes (48%) followed by cellular components (30%), and molecular functions (22%); **(C)** A major category of DEGs fall under cellular processes in which most of the DEGs belongs to cell, cell part, organelle, and protein-containing complex (17%) followed by membrane, membrane part, and extracellular region (8%); **(D)** In biological processes, cellular and metabolic processes (24%), regulation of biological process, and biological reaction (16%), etc. were enriched; **(E)** DEGS categorized under molecular functions were mainly distributed under catalytic activity (50%), binding (33%), transcription regulator activity (11%), and molecular function regulator (6%).

Based on the GO study, the DEGs were categorized into cellular components, biological processes, and molecular functions (Figure 2). A total of 38 genes were categorized under biological processes (47.5%) followed by cellular components (24 genes, 30%) and molecular functions (18 genes, 22.5%). In the biological processes, genes involved in the metabolic process (24%), cellular process (24%), biological regulation (16%), regulation of biological process (16%), signaling (8%), response to stimulus (8%), growth (2%), and cell proliferation (2%) were highly enriched. Similarly, genes associated with catalytic activity (28%), protein-containing complex (12%), organelle (12%), cell part (12%), cell (12%), membrane (6%), membrane part (6%), extracellular regions (6%), organelle part (3%), and extracellular region part (3%) were differentially enriched under cellular components category. In the molecular functions category, binding (67%), transcription regulator activity (22%), and molecular function regulator (11%) were enriched significantly.

### Kyoto Encyclopedia of Genes and Genomes Pathway Analysis of Differentially Expressed Genes

**FIGURE 3:**
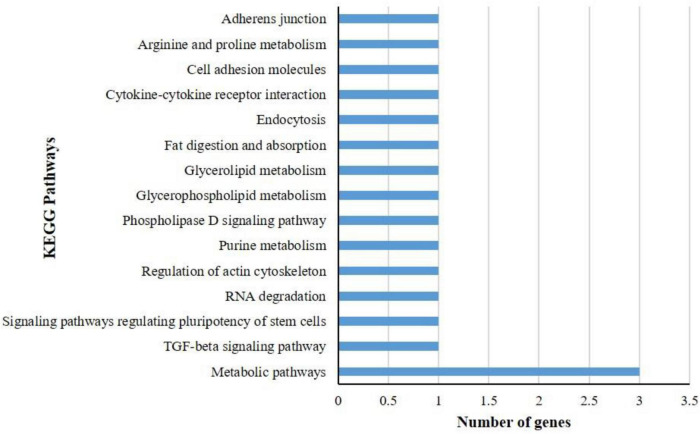
KEGG pathway analysis of differentially expressed genes (DEGs) of *B. tabaci* Asia II 1 at an early stage of ChiLCV infection. KEGG pathway analysis of DEGs showed that the genes were involved in the functions like metabolic pathways, biosynthesis of secondary metabolites, signaling pathways, and actin cytoskeleton regulation in which mostly DEGs were involved in metabolic pathways.

KEGG pathway analysis of differentially expressed genes showed that a total of 17 DEGs in *B. tabaci* were involved in different functions like metabolism, signaling pathways, and cellular processes. The metabolic pathways that were majorly affected by the DEGs were metabolic pathways, TGF-beta signaling pathway, signaling pathways regulating pluripotency of stem cells, RNA degradation, regulation of actin cytoskeleton, purine metabolism, phospholipase D signaling pathway, glycerophospholipid metabolism, glycerolipid metabolism, fat digestion and absorption, endocytosis, cytokine-cytokine receptor interaction, cell adhesion molecules, arginine and proline metabolism, and adherens junction (Figure 3). The pathways identified in GO enrichment analysis were consistent with the findings of the KEGG pathway study.

### Validation of Gene Expression in Reverse Transcription-Quantitative Polymerase Chain Reaction

To validate the differential expression of *B. tabaci* genes in response to ChiLCV infection, 20 highly regulated genes of *B. tabaci* were selected and mRNA expression levels were quantified in RT-qPCR. The primer pairs that produced a single sharp amplicon at the same PCR conditions for endogenous control (β-actin) primers were selected for RT-qPCR assay. Primer pair, AG177F and AG178R (Supplementary Table 1) specific to β-actin produced sharp bands at annealing temperatures of 54–59°C. The annealing temperature of target genes was standardized within the same temperature range. One primer pair for each of the target genes was optimized for RT-qPCR assay (Supplementary Table 1). The relative expression of a target gene was estimated using the 2^–ΔΔ^ *^C^*_*T*_ method (Roy et al., 2021). The C_*T*_ value of β-actin was used to normalize variation among biological replicates. Among the highly regulated genes that were selected for the RT-qPCR assay, the genes like Fas2-like (Bta00469) showed the highest regulation at an early stage of ChiLCV infection. The mRNA expression level of Fas2-like was upregulated by 4.518-fold in the RT-qPCR assay. TLR3 (Bta12916) was another upregulated gene with log_2_ 3.517-fold change in mRNA expression in response to ChiLCV infection. The mRNA expression of replication factor A protein 1 (RFA1) was upregulated by log_2_ 0.403-fold in ChiLCV-exposed *B. tabaci* adults in comparison to non-exposed adults. As a result of exposure to ChiLCV, the expression of GMPR of *B. tabaci* (Bta00655) was elevated by a factor of log_2_ 2.246-fold. Expression of protein Tob1 (Bta07280) was also augmented by log_2_ 1.34-fold at an early stage of ChiLCV infection. Likewise, the mRNA expression levels of a few other genes like AGBL3-like (Bta06925), DUSP10-like (Bta09526), QRSL1 (Bta13949) were elevated by log_2_ 0.168, 2.593, and 0.081-fold, respectively, in the RT-qPCR assay. An uncharacterized protein, CG13607 (Bta13910) was also upregulated by log_2_ 1.74-fold in the ChiLCV-exposed *B. tabaci* population in comparison to non-exposed populations.

Among the highly downregulated genes, expression of *B. tabaci* Actbeta (Bta15272) decreased by log_2_ 5.982-fold in the RT-qPCR assay at an early stage of ChiLCV infection. The next highly downregulated genes in RT-qPCR were TBX20 (Bta05443) and HOXA2 (Bta14472). Expression of TBX20 and HOXA2 declined by log_2_ 3.517, 2.378-fold, respectively in response to ChiLCV infection. The mRNA expression level of NBEAL1 (Bta07414) was also downregulated by log_2_ 1.146-fold in ChiLCV-exposed *B. tabaci* in comparison to non-exposed populations. A log_2_ 0.793-fold downregulation for MRJP (Bta05465) was also recorded in response to ChiLCV infection. Few other genes of *B. tabaci* like ARID (Bta03804), anther specific protein (sf2)-like (Bta01185), plsC (Bta15094) showed significant downregulations by log_2_ 0.241, 0.036, and 1.687-fold, respectively, in RT-qPCR assay post-ChiLCV exposure.

**FIGURE 4:**
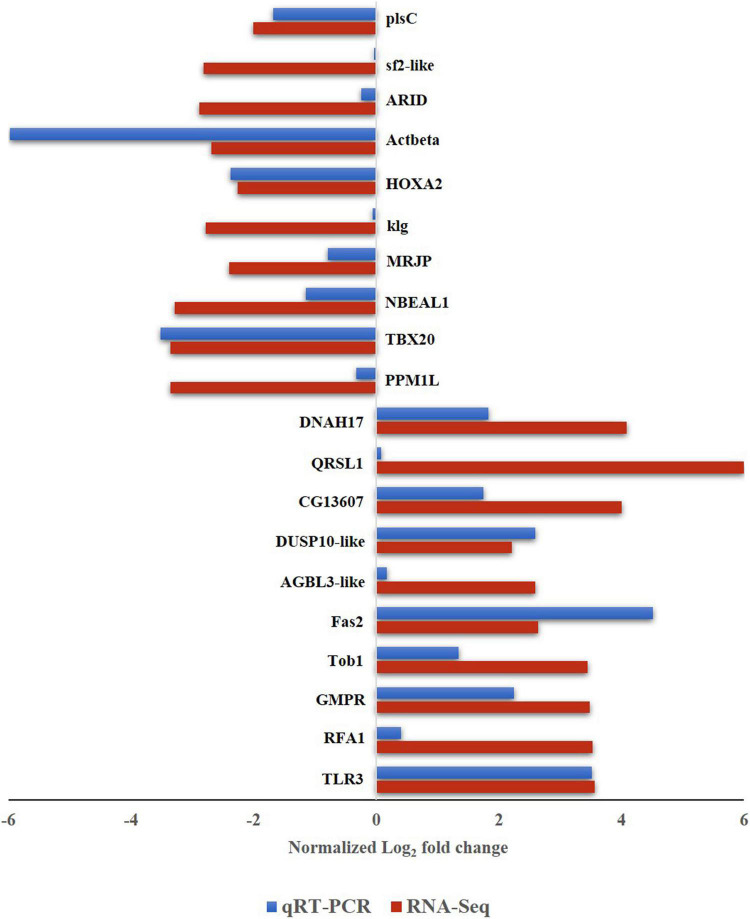
Expression of *B. tabaci* Asia II 1 putative genes in response to ChiLCV infection in RNA-Seq and RT-qPCR. The values of log_2_-fold changes calculated in RNA-Seq analysis were in accordance with the RT-qPCR fold change values.

All the primer pairs for the target and endogenous control genes produced single specific peaks without any secondary amplifications in the RT-qPCR melting curve analysis that indicated the specificity of the reactions. The melting temperatures of RT-qPCR amplicons were listed in Supplementary Table 1. The melt curve of each reaction has been provided as Supplementary Figure 1. The relative expression of selected *B. tabaci* genes in response to ChiLCV infection substantiated the RNA-Seq analysis (Figure 4).

## Discussion

The molecular interactions between *B. tabaci* cryptic species and begomoviruses are not universal (Czosnek et al., 2017) and not much is known about the interaction of ChiLCV with *B. tabaci*. For successful transmission, the begomovirus particles need to reach the salivary glands of *B. tabaci*. It takes 4–7 h after the onset of feeding to be circulated through hemolymph in coated vesicles and translocated into the primary salivary glands (Czosnek et al., 2002). In our recent study, ChiLCV copies were estimated in individual *B. tabaci* at different exposure of active acquisition feeding (Roy et al., 2021). A 6 h sacquisition was found adequate for the successful transmission of ChiLCV by *B. tabaci* Asia II 1 (Senanayake et al., 2012; Roy et al., 2021). About 7.86 × 10^13^ copies of ChiLCV can be acquired by an individual *B. tabaci* adult female during 6 h of feeding on an infected chilli leaf (Roy et al., 2021). Comparison between ChiLCV-exposed and non-exposed *B. tabaci* transcripts post-6 h of acquisition revealed differential expression of 80 DEGs involved in replication factor, cell adhesion receptor, and intracellular trafficking. GO analysis indicated the majority of the DEGs were involved in biological processes followed by cellular components and molecular functions. KEGG pathway analysis showed that the majority of the DEGs were involved in the metabolic pathways of *B. tabaci*.

The key upregulated genes included TLR3, Fas2, GMPR, RFA1, Tob 1, DUSP, DNAH17, AGBL3, and QRSL1. Several genes of *B. tabaci* that were related to immune response pathways such as TLR3, Fas2, RFA1, and GMPR were induced in response to ChiLCV infection. Toll receptors are important parts of the insect’s innate immune system by triggering a cascade of signaling pathways. In mammals, it induces interferons to confer antiviral resistance (Ozato et al., 2002). Toll receptors might have a function in limiting the virus infection in vector and upregulation of TLR3 might be activated upon ChiLCV infection to preclude the adverse effect of the virus on the vector. Another component of *B. tabaci* immunoglobulin (Ig)-related superfamily, Fas2 was upregulated post-exposure to the ChiLCV in the present study. Fas2 belongs to the superfamily of cell adhesion receptors with structural similarity to the vertebrate receptor NCAM (Grenningloh et al., 1990; Brummendorf and Rathjen, 1993). NCAM molecules also have a role in viral attachment to promote virus penetration into the host cells but inhibit the replication of rabies virus *via* induction of interferon β (Hotta et al., 2007), which is mainly involved in innate immunity against viral infection. Silencing Fas 2 using dsRNA results in an increase of ChiLCV copies in *B. tabaci* Asia II 1 (Chakraborty and Ghosh, 2022). This suggests that upregulation of Fas2 transcripts in *B. tabaci* post-ChiLCV infection is due to the innate immune response against the virus infection. GMPR plays an important role in the purine salvage pathway and regulates intracellular purine nucleotides. Besides, it has a role in host innate immunity (Imamura et al., 2020). Upregulation of this gene after ChiLCV exposure indicated induction of defense in *B. tabaci* upon ChiLCV exposure. RFA1 is an ssDNA binding protein (Wold, 1997). Although replication factors play an important role in DNA replication, recombination, and repair (Vanoli et al., 2010; Yamamoto et al., 2019), ChiLCV-DNA does not replicate within the vector (Rosen et al., 2015; Ghosh et al., 2021). This rejected the possibility of involvement of RFA1 in virus multiplication in *B. tabaci*. Although not confirmed, upregulation of RFA1 the ChiLCV-exposure might be due to the employment of innate immune response by *B. tabaci* to protect itself from virus nuclease attack.

Some genes such as Tob1 and DUSP10 having a functional role in the virus lifecycle were also upregulated to favor virus transcription. Gene encoding Tob1 protein was found upregulated in *B. tabaci* post-ChiLCV exposure. Overexpression of Tob1 suppresses cell growth (Shan et al., 2009). The upregulation of this gene in the current study might lead to cellular dysfunction in *B. tabaci* to favor ChiLCV infection. DUSP10, also called MPK5 is involved in the regulation of mitogen-activated protein kinases. These proteins are the major modulators of critical signaling pathways that are dysregulated in various biotic stresses (Patterson et al., 2009). DUSP facilitates vaccinia virus transcription (Liu et al., 1995). Upregulation of this gene indicated the activation of signaling cascades in response to ChiLCV infection and functional role in the virus lifecycle in *B. tabaci*.

Expression of *B. tabaci* putative genes like DNAH17, AGBL3, and QRSL1 was manipulated by ChiLCV to facilitate its circulation within the vector. Several viruses access dynein to mediate the viral replication processes. Silencing of dynein reduced the infection of Murine leukemia virus (Valle-Tenney et al., 2016). Carboxypeptidases play an important role in the stimulation of slow migrating forms of cowpea mosaic virus (CPMV) to fast migrating CPMV leading to increased infectivity (Niblett and Semancik, 1969). Upregulation of DNAH17 and AGBL3 post-ChiLCV exposure in the present study might be due to the manipulation of *B. tabaci* genes by ChiLCV to facilitate the infection and circulation of the virus within the vector. QRSL1 is a highly conserved protein throughout eukaryotes and prokaryotes. This gene is essential for the proper translation of the proteins (Morris et al., 2008). Upregulation of this gene post-ChiLCV exposure might be due to the utilization of host translation apparatus by the virus.

Other than the above-mentioned upregulated genes, a few immune-associated genes like AGO2, PPM1L, NBEAL1, TBX20, and Actbeta were downregulated in *B. tabaci* upon ChiLCV exposure. AGO2 is a major component of the RNA-induced silencing complex (RISC). In *Drosophila*, loss of function of AGO2 leads to susceptibility against Drosophila C virus and Cricket paralysis virus (Van Rij et al., 2006). PPM1L is known to be associated with the replication of viruses like HIV-1, HIV-2, Ebola virus, papovavirus, adenovirus, and rift valley fever virus (Brown et al., 1994; Modrof et al., 2002; Nekhai et al., 2007; Zeng et al., 2009; Baer et al., 2016). Neurobeachin is a peripheral membrane protein of the BEACH domain protein family that is involved in the subcellular targeting of membrane proteins (De Lozanne, 2003). Increased expression of neurobeachin was recorded in stimulated immune cells (Wang et al., 2001, 2004). As an essential factor of autoimmunity, TBX20 produces IgG2a *via* activation of B cells during viral infection (Rubtsova et al., 2013). Inhibin plays an important function in immunological cell development (Licona-Limon and Soldevila, 2007). Lack of plsC activity increased the virulence and infectivity of coxsackievirus (Karlsson et al., 2009). Downregulation of these immune-associated genes in *B. tabaci* post-ChiLCV acquisition indicated a possibility of suppression of vector immune response by ChiLCV to become circulative in its vector.

Transcription regulatory genes like HOXA2, klg, and ARID were also downregulated to support ChiLCV entry and movement within *B. tabaci.* Homeobox proteins are transcriptional factors that regulate several genes in insects (Cillo et al., 2001). In tomatoes, homeobox protein was found to be involved in limiting programmed cell death that limits pathogens (Mayda et al., 1999). In ChiLCV-exposed *B. tabaci*, downregulation of HOXA2 might be due to the suppression activity of ChiLCV that triggered the repression in *B. tabaci*. Klg is a CAM orthologue in insects that is required for photoreceptors and the prevention of excessive synapses (Shimozono et al., 2019). Upon infection of human cytomegalovirus (HCMV), klg is downregulated and causes increased adhesion of the virus to the tumor cells and transendothelial penetration (Blaheta et al., 2004). Downregulation of klg might help the binding and cellular entry of ChiLCV. ARID contains a DNA binding domain (Patsialou et al., 2005) and is an important factor for development, tissue-specific gene expression, and cell growth regulation (Kortschak et al., 2000; Wilsker et al., 2002). This gene is also involved in transcriptional activation and repression of genes through chromatin remodeling (Zhang et al., 2016). Repression of ARID in *B. tabaci* might favor ChiLCV entry and movement within the vector.

Putative genes that code for anther specific protein, major royal jelly protein, and CG13607 were also differentially expressed in *B. tabaci* at an early stage of ChiLCV infection. Although these genes have been characterized in other insects, their functions in *B. tabaci* and begomovirus transmission are yet to be explored. The expression of several orphan genes in *B. tabaci* Asia II 1 was also modulated upon ChiLCV exposure. Functional validation of these orphan genes in *B. tabaci* Asia II 1 and their role in virus transmission need further in-depth study.

## Conclusion

In conclusion, we have assembled a whole-body transcriptome of *B. tabaci* Asia II 1. Several genes of *B. tabaci* associated with innate immune response, cell adhesion, and intracellular trafficking were regulated in response to ChiLCV infection to facilitate its survival and circulation within the vector, *B. tabaci*. The present study helps in understanding the network of molecular interactions between *B. tabaci* Asia II 1 and ChiLCV. Data generated in this study will enrich genomic information of whitefly and will enable future functional studies. The candidate genes of *B. tabaci* that are involved in key physiological processes and ChiLCV transmission would be novel targets for sustainable management of the whitefly-begomovirus complex.

## Data Availability Statement

The datasets generated during the current study are available in the NCBI with BioProject ID PRJNA759071.

## Author Contributions

AG conceived and designed the research and wrote and edited the final manuscript. AN and PC conducted the experiments, recorded the experimental data, and wrote the draft manuscript. AN, PC, MI, and SJ performed the analysis. AG and VB reviewed the data. All authors read and approved the manuscript.

## Conflict of Interest

The authors declare that the research was conducted in the absence of any commercial or financial relationships that could be construed as a potential conflict of interest.

## Publisher’s Note

All claims expressed in this article are solely those of the authors and do not necessarily represent those of their affiliated organizations, or those of the publisher, the editors and the reviewers. Any product that may be evaluated in this article, or claim that may be made by its manufacturer, is not guaranteed or endorsed by the publisher.

## Footnotes

1
https://talk.ictvonline.org/taxonomy/

2
http://www.bioconductor.org/packages/release/bioc/html/DESeq2.html

3
http://geneontology.org/

4
http://www.genome.jp/kegg

5
http://www.whiteflygenomics.org/cgi-bin/bta/blast.cgi
