# Supplementary material for: Transcriptomic Changes of Bemisia tabaci Asia II 1 Induced by Chilli Leaf Curl Virus Trigger Infection and Circulation in Its Vector
Source: Front Microbiol. 2022 Apr 28;13:890807. doi: 10.3389/fmicb.2022.890807 (PMC9096263; doi:10.3389/fmicb.2022.890807)
Supplement: Supplementary file 1 [file Table_1.pdf]

Supplementary Table 1. List of primers used in this study.

S. No.	Gene name	Primer Name	Primer Sequence (5´-3´)	Annealing temperature (°C) in PCR	Amplicon size (bp)	Melting temperature (°C)	Reference
**Primer used for ChiLCV identification in PCR**							
1	Begomovirus DNA-A	Begomo F<br>Begomo R	ACGCGTGCCGTGCTGCTGCCCCCATTGTCC<br>ACGCGTATGGGCTGYCGAAGTTSAGAC	53	2761	-	Akhter et al., 2009
2	ChiLCV coat protein	AG149F<br>AG150R	TGAACAGGCCCATGAACAG<br>ACGGACAAGGAAAAACATCAC	53	290	-	Roy et al., 2021
**Primer used for *B. tabaci* cryptic species identification in PCR**							
3	mtCOI gene	C1-J-2195<br>L2-N-3014	TTGATTTTTTGGTCATCCAGAAGT<br>TCCAATGCACTAATCTGCCATATTA	53	860	-	Simon et al., 1994
**Primers used for gene expression analysis in qRT-PCR**							
4	β-actin	AG177F<br>AG178R	ACATGGAAAAGATCTGGCAT<br>TGAGTCATCTTTTCACGGTT	54-59	121	81	This study
5	Toll receptor 3	AG309F<br>AG310R	CCAAGACCTCTACCTGAGTA<br>GTAATCCGTACAGCTTGCTA	56	152	83.5	This study
6	Dynein heavy chain	AG325F<br>AG326R	CTATGTCTCAAACGAGTGCT<br>AGGTGATTATGGGAGTTGGA	56.5	162	82.9	This study
7	Fasciclin-2	AG283F<br>AG284R	CTGGTGTTTTGACAATCGAC<br>TGATTATGCCTTCTTCCGTC	55	150	74.6	This study
8	Cytosolic carboxypeptidase 3	AG297F<br>AG298R	TAATCACCGGGAGGGTACA<br>CTTGCTCTGAGTCTCCTGTTA	55	100	76.9	This study
9	Tob1 protein	AG301F<br>AG302R	AGGTCAGCTATAGGATTGGT<br>TGAGCTGACTTAAACTGGAC	56	168	81.8	This study
10	CG13607	AG319F<br>AG320R	ACTTCAATCGACAAGTCTCC<br>GGATCCCAGTAGGCAAATAG	58	199	86.3	This study
11	Glutamyl-tRNA(Gln) amidotransferase subunit A	AG323F<br>AG324R	GTATGCCGCTTAACCACC<br>CATTACAAACTCGCCAAAGC	56	197	85.1	This study
12	Dual specificity protein phosphatase 10	AG447F<br>AG448R	CTCGTCTCTTATCAGGGTCT<br>GTAGTGACGATTTCCGACTC	55	142	75.5	This study
13	GMP reductase 1	AG443F	CTATAGATCAGCGGAGGGT	56.2	173	83.3	This study

S. No.	Gene name	Primer Name	Primer Sequence (5´-3´)	Annealing temperature (°C) in PCR	Amplicon size (bp)	Melting temperature (°C)	Reference
		AG444R	GATTTAGCTGTTGAGTGCAT				
14	Replication factor-a	AG315F<br>AG316R	CGCAAACTACCTTTCAGTTC<br>TTCAAGTAACAGGTTGTCCC	55.5	147	74.6	This study
15	T-box transcription factor TBX20	AG237F<br>AG238R	GGACAACAAACGGTATAGGT<br>TCCATCTCGTTGTTGGTAAG	56	187	83.5	This study
16	Anther specific protein	AG227F<br>AG228R	GTCCTCACGAAACCTTTGTA<br>GAAGCATCTTCCTATTCCGA	55.5	194	75.6	This study
17	Major royal jelly protein	AG245F<br>AG246R	CACGTCTTCTCTTCAGACTC<br>ATATTCGTCTTGGAGAACGG	56	198	85.5	This study
18	Inhibin beta chain	AG279F<br>AG280R	GGTACTACGCCAACTACTG<br>GCTTGATGATATTGCTATCCG	56.3	195	74.6	This study
19	Neurobeachin-like protein 1	AG249F<br>AG250R	ATGAACACTTTTTGGGCTG<br>CGTTCATCTGAAACCGTAAT	55	100	79.8	This study
20	Protein phosphatase 1L	AG265F<br>AG266R	AGTGCTGGACTTGTGCACTG<br>ACGGACACACCTAACGGAAG	59	183	82.9	This study
21	AT-rich interactive domain-containing protein	AG233F<br>AG234R	CATATACTTCGGAAGGACCC<br>AAGAAGGTACTTCGCTTACC	59	199	86.3	This study
22	Homeobox protein Hox-A2	AG269F<br>AG270R	TAAGAGCTCCTCGAAAGAAG<br>AGAAGATCACATTGCGCTTG	58.5	132	82.7	This study
23	1-acyl-sn-glycerol-3-phosphate acyltransferase	AG275F<br>AG276R	ATAGACCGTAAAGAACCAGC<br>AGACCATGGGAATGATAGGA	56	190	74.8	This study
24	Klingon	AG453F<br>AG454R	CATCACCCTTGAGAAGGTAG<br>CTTTTCAACCTCGATTTCGG	55	133	83.7	This study

Supplementary Fig. 1. Melting curves of the amplicons in qRT-PCR analysis

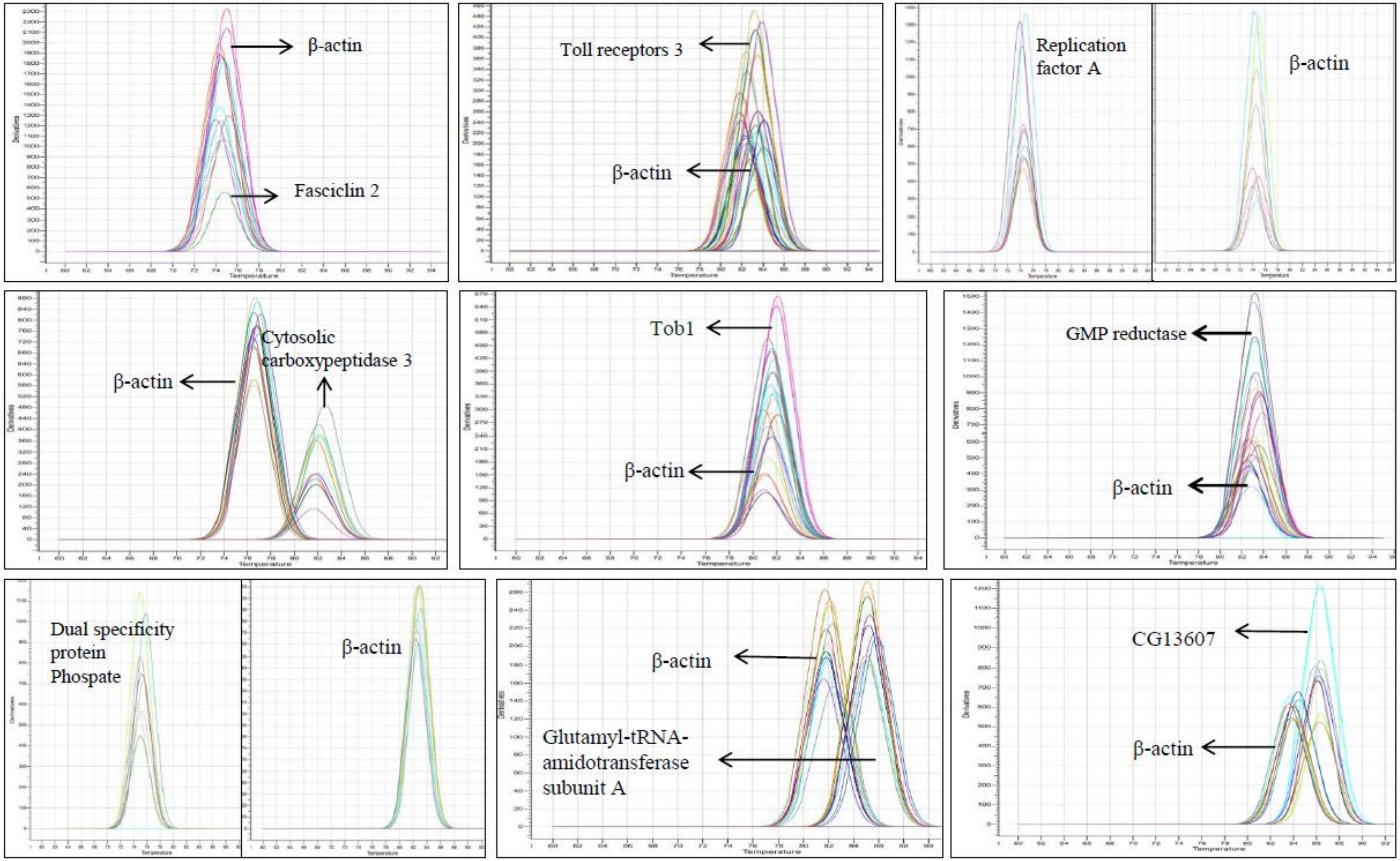

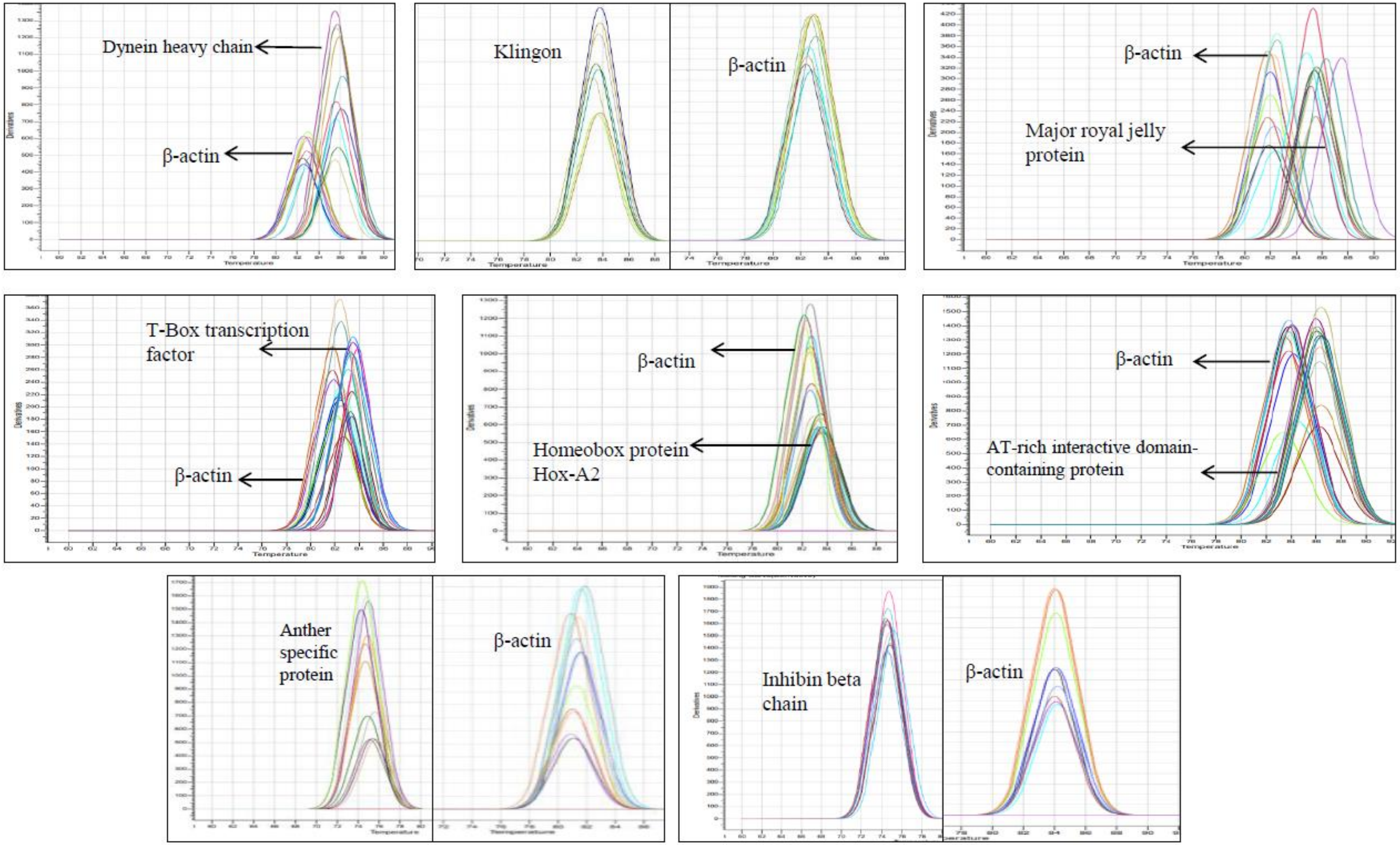

Dynein heavy chain
β-actin
Klingon
β-actin
β-actin
Major royal jelly protein
T-Box transcription factor
β-actin
β-actin
Homeobox protein Hox-A2
β-actin
AT-rich interactive domain-containing protein
Anther specific protein
β-actin
Inhibin beta chain
β-actin

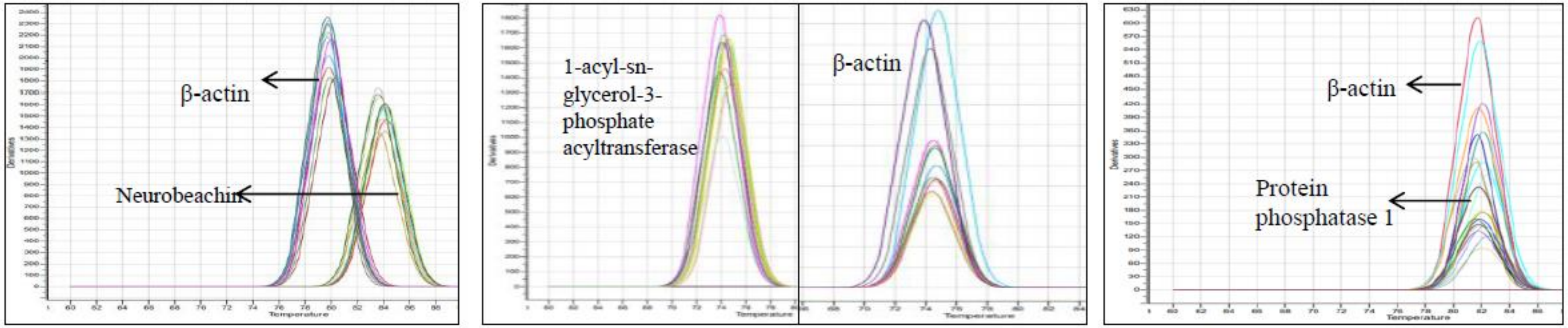
β-actin
Neurobeachin
Temperature
1-acyl-sn-glycerol-3-phosphate acyltransferase
β-actin
Temperature
β-actin
Protein phosphatase 1
Temperature
